# Is Alzheimer's disease an infectious neurological disease? A review of the literature

**DOI:** 10.1002/brb3.2728

**Published:** 2022-07-25

**Authors:** Olivier Uwishema, Ashraf Mahmoud, Jeffrey Sun, Inês F. Silva Correia, Niovi Bejjani, Maria Alwan, Aderinto Nicholas, Adekunbi Oluyemisi, Burhan Dost

**Affiliations:** ^1^ Department of Research and Education Oli Health Magazine Organization Kigali Rwanda; ^2^ Clinton Global Initiative University New York New York USA; ^3^ Faculty of Medicine Karadeniz Technical University Trabzon Turkey; ^4^ Faculty of Medicine Kilimanjaro Christian Medical University College Moshi Tanzania; ^5^ Faculty of Health Sciences McMaster University Hamilton Ontario Canada; ^6^ School of Medicine, Faculty of Health, Medicine, Education and Social Care Anglia Ruskin University Chelmsford UK; ^7^ Faculty of Medicine University of Saint Joseph of Beirut Beirut Lebanon; ^8^ Faculty of Medicine Beirut Arab University Beirut Lebanon; ^9^ Department of Medicine and Surgery Ladoke Akintola University of Technology Ogbomosho Nigeria; ^10^ School of Public and Allied Health Babcock University Ilishan‐Remo Nigeria; ^11^ Department of Anesthesiology, School of Medicine Ondokuz Mayis University Samsun Turkey

**Keywords:** Alzheimer's disease, microorganism, neuroinflammation, neurology

## Abstract

**Introduction:**

Alzheimer's disease (AD) is a leading cause of dementia around the globe. Its pathogenesis is characterized primarily by the extracellular deposition of amyloid β peptides and intracellular neurofibrillary tangles. Despite the significant investments in neurological research, the exact molecular mechanism of AD pathogenesis is still not fully elucidated. Several studies converge on a hypothesis that pathogenic microbes might play a role in AD progression. Although this hypothesis has been considered relatively weak for decades, it has recently received considerable attention due to increasing evidence on the association between microorganisms and AD. There is a lack of experimental and scientific arguments conveying that these microorganisms engender cognitive and neuropathological deficits and modifications specific to AD, challenging the theory that it could be an infectious neurological disease. This review focuses on recent advances in the infection hypothesis and provides an overview of new findings portraying the significance of pathogenic microbes in AD and the challenges confronting the validity of the hypothesis.

**Methodology:**

Data were collected from medical journals published on PubMed, Ovid MEDLINE, ScienceDirect, and Embase bibliographical databases with a predefined search strategy. All articles considering neurological disorders, especially AD associated with infectious diseases, were included.

**Results:**

This work focused on providing an overview of new findings around the relationship between microorganisms and AD, challenges facing the validity of the theory, and recommendations on how the scientific community can best develop alternative approaches to address the pathophysiology of AD.

**Conclusion:**

While many studies reinforce the suspicion of an infectious etiology of AD, it is important to note that it is yet not validated how microorganisms’ presence in the brain can develop AD due to the limited available evidence. Certainly, ground‐breaking work is mandatory in this field of research, and these reports so far warrant a thorough investigation into how a chronic infection may remain silent while progressing its neuroinflammation. Amid this uncertainty arises the hope that many researchers will take on this challenge and join this endeavor to benefit AD patients worldwide.

## Graphical Abstract

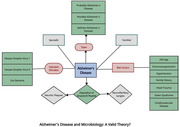

## 1 INTRODUCTION

Alzheimer's disease (AD) is a neurodegenerative disease characterized by neuritic plaques and neurofibrillary tangles from amyloid‐β peptide A build‐up in the medial temporal lobe and neocortical areas of the brain (De‐Paula et al., 2012). The disease clinically presents with a slow progression of cognitive and behavioral impairment. Approximately 50 million people across the globe suffer from the most common manifestation of the disease—dementia—and this figure is expected to double every 5 years, with an estimated 152 million cases by the year 2050 (Livingston et al., 2020; Yiannopoulou & Papageorgiou, 2020). What is more alarming is that both the cause and cure for AD have not yet been well understood and developed, respectively.

Clinical diagnostic criteria for the disease were developed in 1984 by the Alzheimer's Disease and Related Disorders Association and the National Institute of Neurological and Communicative Disorders and Stroke study group. The criteria include the following:

1. Probable Alzheimer's disease: identified by dementia diagnosis through neuropsychological testing, progressive amnesia, and impaired activities of daily living (McKhann et al., 1984; Neugroschl & Wang, 2011);
2. Possible Alzheimer's disease: devoid of neurologic and psychiatric disorders, and comorbidities, such as a systemic or brain illness; can also induce dementia, although not the primary cause (McKhann et al., 1984; Neugroschl & Wang, 2011);
3. Definite Alzheimer's disease: confirmed by histopathological testing. (McKhann et al., 1984; Neugroschl & Wang, 2011)

This commentary aims to provide an overview of new findings around the relationship between microorganisms and AD, challenges facing the validity of the theory behind this relationship, and recommendations on how the scientific community can best develop alternative approaches to address the pathophysiology of AD.

## 2 THE CHARACTERISTICS OF AD

### 2.1 Prevalence

AD is the principal cause of dementia in older individuals as its prevalence rises with age. In individuals from 65 to 74 years of age, the incidence is about 3%; those between 75 and 84 years of age have a 19% incidence, and those above 84 years of age have a 47% incidence (Kumar et al., 2017). Alzheimer's cases are mostly sporadic, with 5%–10% being hereditary forms, seen at an earlier onset (Kumar et al., 2017).

### 2.2 Mechanism

AD results from the intracranial build‐up of two proteins, Aβ and tau, forming extracellular neuritic plaques and intracellular neurofibrillary tangles, respectively. This results in neuronal dysfunction and death, and inflammatory responses. When amyloid precursor protein (APP) is cleaved by β‐amyloid‐converting enzyme and γ‐secretase instead of α‐secretase and γ‐secretase, it releases Aβ peptides, which culminate in the accumulation of plaques and tangles (De‐Paula et al., 2012).

### 2.3 Risk factors

Multiple loci have been associated with the increased generation and deposition of Aβ protein, such as mutations in components of γ‐secretase (encoded by the presenilin 1 or 2 genes), or mutations in the APP gene on chromosome 21. Population with an extra copy of the APP gene, including patients with Down Syndrome or with small interstitial duplications of the gene, present at higher risk (Kumar et al., 2017). Moreover, the apolipoprotein E ε4 allele on chromosome 19 is responsible for approximately one fourth of the risk of developing late‐onset AD (Kumar et al., 2017).

Despite genetic predisposition, age‐related mechanisms are critical for AD development, whereas—even in genetically susceptible and immunosuppressed individuals (Uwishema et al., 2022)—the illness does not manifest itself until later in life (Castellani et al., 2010). Other risk factors include hypertension, diabetes, hyperlipidemia, cerebrovascular diseases (Hall, 2015), family background of dementia, head trauma, female sex, poor education level, and environmental factors such as toxic metals, insecticides or pesticides, industrial or commercial waste, antimicrobial agents, and air impurities (Castellani et al., 2010).

## 3 NEW FINDINGS ON PATHOGENIC MICROBES IN AD

It has been hypothesized that the infectious herpes simplex virus 1 (HSV‐1) could play a role in the development of AD since its DNA could affect the brain tissue in a significant proportion of elderly individuals. Despite a lack of scientific arguments, these findings were frequently faced with mockery, and hatred, or were simply ignored (Itzhaki et al., 2020). According to a recent study, HSV‐1 has been shown to cause β‐amyloid peptide synthesis and an increase in tau phosphorylation (Powell‐Doherty et al., 2020). In cell and animal models, HSV‐1 causes β‐amyloid peptide aggregation, the creation of senile plaques, and the production of neurofibrillary tangles. Moreover, HSV‐1 produced hyperexcitability and elevated intracellular calcium signals in rat cortical neurons, leading to a change in the amyloid precursor processing pathway and an aberrant increase in amyloid‐β formation (Ekundayo et al., 2021).

There is evidence that HSV can cause β‐amyloid peptide (A40 and A42) synthesis in human neuroblastoma cells. Furthermore, Alzheimer's patients’ frontal and temporal cortex and hippocampus have been recently found to have human herpes virus 6 (HHV‐6) (Santpere et al., 2020). This virus may create lesions in the infected patient's brain, although not directly connected to AD (Ekundayo et al., 2021).

Every healthy person has a unique gut microbiota community of bacteria that is symbiotic. The gut microbial community includes *Lactobacillus species*, *Bifidobacteria* (*Actinobacteria*), *Verrucomicrobia*, *Spirochetes*, *Proteobacteria*, *Fusobacteria*, *Firmicutes*, and *Cyanobacteria*. Intestinal bacteria are required for homeostasis and protection against harmful pathogens (Wu & Wu, 2012). Dysbiosis of the gut microbiota has been related to extraintestinal diseases such as neurological disorders (Faruqui et al., 2021) with recent studies suggesting that gut bacteria may have a role in aggravating the development of AD (Faruqui et al., 2021; Wu et al., 2021).

## 4 CHALLENGES FACING THE THEORY VALIDITY

Recent studies support the theory of an infectious etiology of AD, but the available evidence is limited and, in some studies, inconsistent (Robinson et al., 2004; Vigasova et al., 2021). Thus, this new theory cannot be fully validated due to the many challenges researchers face in this field.

First, most research in this area supports individual organisms but there is also evidence for polymicrobial etiology (Alonso et al., 2017; Alonso et al., 2018; Bu et al., 2014; Carrasco et al., 2020; Carter, 2017; Miklossy, 2008; Pisa et al., 2017; Pisa et al., 2018). Second, various bacteria and fungi associated with AD have also been detected in disease‐free control subjects, suggesting that other factors, besides microorganisms, might be playing a role in the development of the condition (Alonso et al., 2018; Alonso et al., 2017). There is also a lack of experimental evidence showing that microorganisms elicit the cognitive deficits and the neuropathological modifications characteristic of AD (Robinson et al., 2004). Therefore, detecting organisms in the brains of AD patients is not enough to prove that it is an infectious disease (Fulop et al., 2018).

Furthermore, it has been suggested that senile plaques found in AD are biofilms, though the composition and the origin of the amyloids contained in those plaques have not yet been determined (Fulop et al., 2018; Miklossy, 2016). In addition to this, regardless of the advances in antiretroviral therapy, Human Immunodeficiency Virus (HIV) is still known for producing neurological problems in AIDS patients due to its neuroinvasive, neurotropic, and neurovirulent nature (Uwishema et al., 2022). The course of development of a recognizable neurological condition in an HIV‐infected person is determined by an epidemiological triangle consisting of numerous properties of HIV, genetic qualities of the host, as well as interactions with the environment, including treatment methods (Uwishema et al., 2022). Furthermore, even if they are treated, those living with HIV are at a high risk of chronic inflammation. Their immune cells are constantly stimulated, and the primoinfection causes a long‐term lesion of the gut mucosa, culminating in a mild chronic inflammatory response. (Uwishema et al., 2022) For all these reasons, the infectious theory of AD cannot be fully validated.

## 5 CONCLUSION

While many studies reinforce the suspicion of an infectious etiology of AD, it is important to note that it is yet not been validated how microorganisms’ presence in the brain can develop AD due to the limited available evidence.

Certainly, ground‐breaking work is mandatory in this field of research, and these reports so far warrant a thorough investigation into how a chronic infection may remain silent while progressing its neuroinflammation. Amid this uncertainty arises the hope that many researchers will take on this challenge and join this endeavor to benefit AD patients worldwide. A tool for early detection of numerous infections—viral, fungal, and bacterial in origin—could be a potential outcome of further research in this field to avoid the late manifestation of AD in those predisposed to the infections.

## CONFLICT OF INTEREST

The authors declare no conflict of interest.

## AUTHOR CONTRIBUTIONS

Olivier Uwishema conceptualized the study, administered the project, and reviewed and designed the manuscript. Ashraf Mahmoud performed data collection and assembly. Olivier Uwishema reviewed and edited the first draft of the manuscript. Jeffrey Sun reviewed and edited the second draft of the manuscript. Inês F Silva Correia reviewed and edited the third draft of the manuscript. Burhan Dost reviewed and edited the final draft of the manuscript. The figure was drawn and analyzed by Adekunbi Oluyemisi, Olivier Uwishema, Ashraf Mahmoud, and Inês F. Silva Correia. All authors wrote the manuscript and gave final approval for publication.

### PEER REVIEW

The peer review history for this article is available https://publons.com/publon/10.1002/brb3.2728.

## Data Availability

No data have been shared.
